# Effect of Astaxanthin Supplementation on Salivary IgA, Oxidative Stress, and Inflammation in Young Soccer Players

**DOI:** 10.1155/2015/783761

**Published:** 2015-06-18

**Authors:** Ivana Baralic, Marija Andjelkovic, Brizita Djordjevic, Nenad Dikic, Nenad Radivojevic, Violeta Suzin-Zivkovic, Sanja Radojevic-Skodric, Snezana Pejic

**Affiliations:** ^1^Sports Medicine Association of Serbia, Lazarevacki Drum 14, 11000 Belgrade, Serbia; ^2^Institute for Bromatology, Faculty of Pharmacy, University of Belgrade, Vojvode Stepe 450, 11000 Belgrade, Serbia; ^3^Institute of Histology and Embryology, School of Medicine, University of Belgrade, Dr Subotica 1, 11000 Belgrade, Serbia; ^4^Institute of Pathology, School of Medicine, University of Belgrade, Dr Subotica 1, 11000 Belgrade, Serbia; ^5^Snezana Pejic, Department of Molecular Biology and Endocrinology, “Vinca” Institute of Nuclear Sciences, University of Belgrade, P.O. Box 522, 11000 Belgrade, Serbia

## Abstract

The physiologic stress induced by physical activity is reflected in immune system perturbations, oxidative stress, muscle injury, and inflammation. We investigated the effect of astaxanthin (Asx) supplementation on salivary IgA (sIgA) and oxidative stress status in plasma, along with changes in biochemical parameters and total/differential white cell counts. Forty trained male soccer players were randomly assigned to Asx and placebo groups. Asx group was supplemented with 4 mg of Asx. Saliva and blood samples were collected at the baseline and after 90 days of supplementation in preexercise conditions. We observed a rise of sIgA levels at rest after 90 days of Asx supplementation, which was accompanied with a decrease in prooxidant-antioxidant balance. The plasma muscle enzymes levels were reduced significantly by Asx supplementation and by regular training. The increase in neutrophil count and hs-CRP level was found only in placebo group, indicating a significant blunting of the systemic inflammatory response in the subjects taking Asx. This study indicates that Asx supplementation improves sIgA response and attenuates muscle damage, thus preventing inflammation induced by rigorous physical training. Our findings also point that Asx could show significant physiologic modulation in individuals with mucosal immunity impairment or under conditions of increased oxidative stress and inflammation.

## 1. Introduction

Astaxanthin (Asx) is a red-orange carotenoid mainly produced by micro- and macroalgal species and accumulated in many marine organisms, such as shrimps, crabs, trout, and salmon. The polyene system gives astaxanthin its distinctive molecular structure, chemical properties, and light-absorption characteristics [1]. The presence of the hydroxyl and keto moieties on each ionone ring explains some of its unique features such as higher antioxidant (AO) activity, a more polar nature than other carotenoids, and ability to be esterified [2]. Its both high AO potency and polar properties, make Asx an attractive nutraceutical for promising applications in human health and nutrition. Asx has also been attributed with extraordinary potential for protecting the organism against a wide range of diseases [3].

The mucosal immune system functions as the first line of defense against pathogen invasion by preventing the attachments of infectious agents to mucosal surfaces [4]. An important activity of the epithelia lining in gastrointestinal, respiratory, and genitourinary tract is the production of secretory IgA (sIgA), the antibody that largely dominates mucosal humoral immunity [5]. sIgA protects mucosal surfaces by directly cross-linking environmental microorganisms or macromolecules, thus preventing their contact with the surface of epithelial cells and hence facilitating their elimination [6]. Numerous studies of saliva composition have found a decreased sIgA secretion with age [7], psychological, and occupational stresses [8] and also in nutritional deficiencies [9]. In addition, mounting evidence indicates that prolonged and intensive physical exertion can cause a decrease in sIgA concentration and secretion rate [10]. Lowered concentrations of sIgA are associated with an increased frequency of upper respiratory tract infection (URTI) episodes or with reduced protection against certain infections [11].

Intensive and sustained physical activity elevates the generation of free radicals and reactive oxygen species (ROS), thus creating an imbalance between ROS and antioxidants and leading to oxidative stress that not only causes lipid peroxidation and protein oxidation but also may have a negative impact on immune function [12]. Asx possesses antioxidant, free radical scavenging, and anti-inflammatory properties that may affect human immune system and resistance to pathogens, although most data come from* in vitro* and animal studies [13]. These studies showed that Asx stimulated a delayed-type hypersensitivity (DTH) response, the natural killer (NK) cells cytotoxic activity, and increased concentrations of IgG and IgM and B cell population [14–17].

No published human studies exist regarding the influence of Asx ingestion on exercise-induced mucosal immunity dysfunction. One human study showed that Asx could enhance immune response and decrease a DNA oxidative damage and inflammation in healthy females [18]. Therefore, the purpose of the present investigation was to test the hypothesis that Asx supplementation is effective in enhancing sIgA secretion in young soccer players. Compared with previous research in this field, particular strength of this study is the fact it was conducted during a regular competitive season, reflecting habitual conditions of nutrition and training program. In addition, we determined oxidative status parameters, along with biochemical and hematological profile, in order to examine possible connections among mucosal immunity, oxidative stress, inflammation, and Asx supplementation in young, healthy athletes.

## 2. Material and Methods

### 2.1. Subjects

Forty trained male soccer players, among young selection of soccer club “Partizan,” Belgrade, were recruited as experimental subjects. The participants met the following exclusion criteria: smokers, subjects with recurrent respiratory infections, subjects taking antibiotics, subjects taking immunosuppressive drugs, and alcohol abuse. Athletes as well as their parents gave the written consent after having been explained the purpose, demands, and possible risks associated with the study. The study was conducted according to the guidelines laid down in Declaration of Helsinki. Experimental procedures were approved by the Ethical Committee of Sports Medicine Association of Serbia. They were involved in a controlled training program during a 90-day period over competitive season, in which they had 5 to 7 training sessions per week with an average weekly training of 10 to 15 hours and took participation in national championship. They also had different aspects of trainings, including strength, resistance, cardio, flexibility, and proprioceptive training.

### 2.2. Research Design and Supplementation

Soccer players were randomly assigned to Asx (*N* = 21) or placebo (P, *N* = 19) groups. Under double-blind procedures, subjects received Asx (4 mg per day) or placebo supplements for 90 days. The Asx used in this study was a homogenized and spray dried biomass of the green unicellular alga* Haematococcus pluvialis*. Both Asx and placebo capsules were manufactured by Bioreal (Sweden) and generously donated by Oriflame, Serbia. The dietary supplementation comprised of one capsule daily taken orally in conjunction with a meal.

Two weeks before the first test session, subjects reported to the laboratory for orientation, measurement of body composition, and cardiorespiratory fitness. Body weight and total body fat were measured using the BC-418, 8-contact electrode device (Tanita, Tokyo, Japan), which uses bioelectrical impedance analysis for body composition analysis. Height was measured to the nearest 0.1 cm with a portable stadiometer. Body mass index (BMI) was calculated by dividing the weight in kilograms by the square of the height in meters (kg/m^2^). VO_2max_ was measured on a motor driven treadmill (Run race, Techno gym, Italy), using an indirect calorimetry system (Quark b2, Cosmed, Italy) during an incremental exercise test to volitional fatigue. Basic demographic and training data were obtained through a questionnaire. Subjects were instructed to restrain themselves from making any drastic changes in the diet. They agreed to avoid the use of vitamin/mineral supplements, antioxidant supplements, nutritional supplements, ergogenic aids, herbs, and medications known for their effect on immune function, 1 month before and during the study. Subjects recorded food intake in a 4-day food record before the first exercise test session. The food records were analyzed using CRON-O-Meter v0.9.6. software.

Saliva and blood samples were collected at the onset of the study and after 90 days of supplementation, between 9:00 and 10:00 a.m., after overnight fast, before training session. For all collection points, players were restrained from water consumption 10 min before sampling. The training sessions were carried out under the same conditions, in the same place and at the same time of the day to avoid circadian variations. Each subject served as self-control to eliminate any biological variability in the response to Asx supplementation.

### 2.3. Salivary Flow Measurement

Whole unstimulated saliva was collected using salivettes (Sersted, Vumbrecht, Germany), by placing the cotton swab under the tongue. All saliva collections (2 min, electronically timed) were made after players had been sitting quietly for a few minutes, leaning forward, with their heads tilted. Immediately after collection, the obtained saliva samples were separated from the cotton by centrifuging at 1500 ×g for 15 minutes. The supernatant fluid was frozen at −80°C for later analysis of sIgA. Before and after saliva collection, salivettes were weighted on an analytical balance. The amount of saliva in grams was converted to milliliters assuming that the specific gravity of saliva is 1 and divided by 2 to express salivary flow in mL/min.

### 2.4. Measurement of sIgA Concentration and Secretion Rate

The sIgA concentration was measured by enzyme-linked immunosorbent assay (ELISA). Briefly, the test was performed on 96-well microtiter plates, which were coated with goat anti-human IgA (1 : 100 dilution, AbD Serotec, Oxford, UK) in coating buffer (0.1 M carbonate-bicarbonate, pH 9.5) and kept overnight at 4°C. On the day of analysis, plates were incubated for 60 min at room temperature before being washed with TTBS buffer (Tween 20 Tris Buffered saline, pH 7.5) and then blocked with 3% gelatin in TTBS for 2 h at room temperature. Samples were thawed and then diluted (1 : 200) with a sample diluent (1% gelatin in TTBS). Known concentrations of human secretory IgA (AbD Serotec, Oxford, UK) and controls were used to construct a standard curve. The plate was then washed before standards and samples were added to wells (in duplicate) and incubated for 1 h at room temperature. The plate was washed again before addition of diluted goat anti-human horseradish peroxidase conjugates (AbD Serotec, Oxford, UK). After incubation, the plate was washed again before addition of substrate tetramethylbenzidine (AbD Serotec, Oxford, UK) and incubated for 30 min in the dark. The colorimetric reaction was stopped via addition of 100 *μ*L of sulfuric acid, and the absorbance of solution in each well was determined at 450 nm by using ELx800 Absorbance Microplate Reader (Biotek, Winooski, USA). Regression analysis using the relation of standard sIgA concentrations and amount of absorbance (nm) was used to interpolate the concentration of sIgA in the samples. sIgA is expressed as absolute concentration and secretion rate. sIgA secretion rate (*μ*g/min) was determined by multiplying the absolute sIgA concentration (*μ*g/mL) with saliva flow rate (mL/min).

### 2.5. Oxidative Stress Status

For determination of oxidative status parameters, the venous blood was collected into heparin evacuated tube (Greiner Bio-one, Kremsmünster, Austria). The blood samples were centrifuged at 1500 g for 10 min, and aliquots of plasma were stored at −80°C for later analysis.

Total antioxidant status (TAS) was determined using an automated method developed by Erel [19]. A standardized solution of Fe^2+^–o-dianisidine complex reacts with a standardized solution of H_2_O_2_ by a Fenton-type reaction, producing hydroxyl radicals (OH^∙^). These potent ROS oxidize the reduced colorless o-dianisidine molecules to yellow-brown colored dianisidyl radicals at low pH. Antioxidants in the sample suppress the oxidation reactions and color formation. This reaction can be monitored by spectrophotometry. The reaction rate is calibrated with Trolox (a water-soluble analogue of vitamin E, 6-hydroxy-2,5,7,8-tetramethylchroman-2-carboxylic acid) and was incorporated into Ilab 300 plus autoanalyser (Instrumentation Laboratory, Milan, Italy). The TAS value of the samples tested is expressed as mmolTrolox equiv./L.

Total oxidant status (TOS) was determined according to Erels' method [20]. This assay is based on the oxidation of ferrous ion to ferric ion in the presence of various oxidant species in serum. The ferric ion makes a colored complex with xylenol orange in an acidic medium. The color intensity, which can be measured spectrophotometrically, is related to the total amount of oxidant molecules present in the sample. The assay was calibrated with H_2_O_2_ and incorporated into Ilab 300 plus autoanalyser (Instrumentation Laboratory, Milan, Italy). The results are expressed in terms of micromolar hydrogen peroxide equivalent per liter (*μ*molH_2_O_2_ equiv./L).

Prooxidant-antioxidant balance (PAB) was measured according to a previously published method [21]. This method is based on two different oxidation-reduction reactions which take place simultaneously. In enzymatic reaction, the chromogen TMB is oxidized to a colored cation by peroxides, and in the chemical reaction, the colored TMB cation is reduced to a colorless compound by antioxidants. The photometric absorbance is then compared with the absorbance given by a series of standard solutions that are made by mixing varying proportions (0–100%) of 250 mmol/L H_2_O_2_, as a representative of hydroperoxides, which is an indicator of total oxidant status, with 3 mmol/L uric acid (in 10 mmol/L NaOH), as a representative of the antioxidant capacity. This photometric comparison was carried out using an ELISA reader.

TMB powder (60 mg) was dissolved in 10 mL of DMSO. For preparation of TMB cation, 400 *μ*L of TMB/DMSO was added in 20 mL of acetate buffer (0.05 M, pH 4.5), and then 70 *μ*L of fresh chloramine T (100 mM) solution in distilled water was added. The mixture was incubated in a dark place for 2 hr at room temperature, after which 25 units of peroxidase enzyme solution were added into 20 mL TMB cation, dispensed in 1 mL, and put at −20°C. To prepare the TMB solution, 200 *μ*L of TMB/DMSO was added into 10 mL of acetate buffer (0.05 M, pH 5.8). The working solution was prepared by mixing 1 mL of TMB cation with 10 mL of TMB solution. The mixture was incubated for 2 min at room temperature, in a dark place, and it was used immediately. The sample (10 *μ*L), standard, or blank (distilled water) were mixed with 200 *μ*L of working solution, in each well of a 96-well plate, which was then incubated in a dark place at 37°C for 12 min. At the end of incubation time, 100 *μ*L of 2N HCl was added into each well and measured in ELISA reader at 450 nm, with a reference wavelength of 620 or 570 nm (ELx800 Absorbance Microplate Reader, Biotek, Winooski, USA). A standard curve was provided from the values relative to the standard samples. The PAB values of the unknown samples were calculated using the standard curve and expressed in arbitrary HK units, which represent the percentage of H_2_O_2_ in standard solution, multiplied by 6.

### 2.6. Hematological and Biochemical Parameters

Hematological analysis was performed in the blood samples collected into K-EDTA tubes (Greiner Bio-one, Kremsmünster, Austria), using an automated hematology analyzer Cell-Dyn 3700 (Abbott Diagnostics, Illinois, USA). To examine biochemical parameters, blood samples were drawn from an antecubital vein into sample tube with serum separator gel (Greiner Bio-one, Kremsmünster, Austria) and then left to coagulate for 30 min to separate the serum, and the obtained samples were stored at −80°C for later analysis. The following items were included in the general biochemical examination: aspartate aminotransferase (AST), alanine aminotransferase (ALT), creatine kinase (CK), lactate dehydrogenase (LDH), uric acid (UA), creatinine (Cre), high sensitivity C-reactive protein (hs-CRP), total cholesterol (CHOL), HDL cholesterol (HDL-C), and triglycerides (TG). Biochemical analysis was performed using an ILab 300 Plus autoanalyser (Instrumentation Laboratory, Milan, Italy) employing commercial kits (Bioanalytica, Belgrade, Serbia). The concentration of LDL cholesterol (LDL-C) was calculated using the formula of Friedewald et al. [22].

### 2.7. Statistical Analysis

Statistical analyses were performed using the PASW Statistics version 18.0 and MedCalc (version 11.4 Software, Belgium) software. All data were assessed for normality (Kolmogorov-Smirnov tests). The results are expressed in terms of mean values and standard error (SE) for normally distributed variables. Where distribution differed from a normal distribution, geometric means and 95% confidence intervals were given. Subjects' baseline characteristics and nutritional parameters between treatment groups were compared using independent-sample* t*-test. The effects of supplementation and training were analyzed by two-way analysis of variance (ANOVA) with repeated measures to test for the two main effects and for the interaction between them. When a significant *p* value was obtained, Bonferroni* post hoc* comparison test was employed to determine the differences between groups. Two-tailed *p* values are given throughout. Due to the fact that the distributions of sIgA absolute concentrations and secretion rate, AST, ALT, CK, and hs-CRP were skewed, logarithmic transformation of these values was performed before statistical comparisons were made.

## 3. Results

### 3.1. Subjects Characteristics

Physical characteristics for the 40 soccer players randomized to Asx and P groups are summarized in Table 1. No significant differences were found between groups according to age, body composition, or VO_2max_ (*p* > 0.05,* t*-test).

According to 4-day food records analysis using CRON-O-Meter v0.9.6 software, estimated daily energy and nutrient intake in two experimental groups were similar (*p* > 0.05), Table 2.

### 3.2. Salivary Flow, sIgA Absolute Concentration, and Secretion Rate

**Figure 1:**
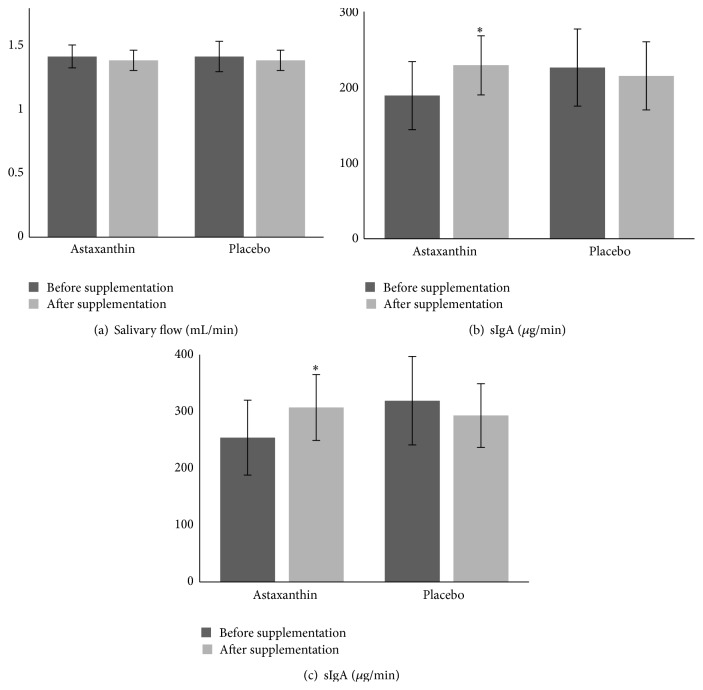
Salivary flow (a), salivary IgA concentration (b), and salivary IgA secretion rate (c) in soccer players at baseline and after 90 days of supplementation. Values are presented as mean ± SE. The difference in relation to baseline was significant at 0.05 (*∗*).

Salivary flow measurement was unaffected by training or supplementation (*p* > 0.05). The obtained values were unchanged after 90 days of training and supplementation (Figure 1(a)).

ANOVA repeated measures showed a significant interaction effect of supplementation and training (*F* = 6.221, *p* < 0.05) on sIgA absolute concentration as well as on sIgA secretion rate (*F* = 4.608, *p* < 0.05) (Figures 1(b) and 1(c)).* Post hoc* comparison revealed a significant increase of sIgA absolute concentration and secretion rate in supplemented group after 90 days when compared to baseline values (*p* < 0.05), while there were no significant changes in P group.

### 3.3. Oxidative Stress Status

Oxidative stress status is reported in Table 3. Regular soccer training had a significant effect on TOS, regardless of supplementation (main effect of training, *F* = 108.678, *p* < 0.001). TOS decreased in both, the supplemented and the P group, after 90 days of regular training in comparison to baseline values. Basal TAS was unaffected by soccer training and supplementation (*p* > 0.05). There was a significant decrease in PAB in Asx group after 90 days in comparison to baseline values (interaction effect of supplementation and training: *F* = 25.371, *p* < 0.05; main effect of training: *F* = 4.205, *p* < 0.001), as a result of continuous training and supplementation. No significant changes were found in P group.

### 3.4. Hematological Parameters

**Figure 2:**
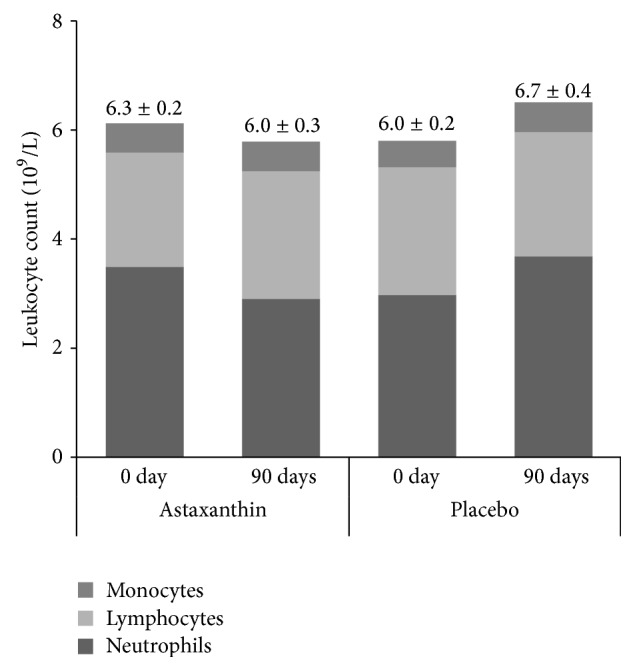
Total leukocyte count (denoted by numbers at the top of the bars); neutrophil, lymphocyte, and monocyte counts in soccer players at baseline and after 90 days of supplementation.

After 90 days of training and supplementation, ANOVA analysis showed a significant increase in leukocyte count in P group (interaction effect of supplementation and training: *F* = 4.528, *p* < 0.05; main effect of training: *F* = 3.989, *p* < 0.05) but not in Asx group, as presented in Figure 2. There was a significant increase in neutrophil count in P group after 90 days of observational period compared to baseline values (main effect of training: *F* = 4.875, *p* < 0.01). These changes were not detected in supplemented group.

### 3.5. Biochemical Parameters

Biochemical parameters are shown in Table 4. ANOVA revealed a significant main effect of training on AST (*F* = 57.029, *p* < 0.001), CK (*F* = 29.000, *p* < 0.01), and LDH (*F* = 87.641, *p* < 0.001) plasma levels.* Post hoc* comparison showed a significant decrease in CK activity in Asx group after 90 days compared to baseline values (*p* < 0.01), while a decrease in P group was not statistically significant. After 90 days of continuous training, AST and LDH decreased significantly in both Asx (*p* < 0.001, *p* < 0.01, resp.) and P group (*p* < 0.01, *p* < 0.5, resp.). In addition, a significant main effect of supplementation was observed on AST (*F* = 3.979, *p* < 0.05) and LDH (*F* = 3.995, *p* < 0.05) levels, with lower activity in Asx compared to P group.

UA and Cre levels significantly decreased in both groups after 90 days of observational period (UA main effect of training, *F* = 5.528, *p* < 0.05; Cre main effect of training, *F* = 4.429, *p* < 0.05). ANOVA analysis also showed a significant supplementation and training interaction effect (*F* = 4.050, *p* = 0.05) on hs-CRP levels. Namely, after 90 days of regular soccer training, a 57% increase in hs-CRP levels may be observed in P group, but not in the Asx group. Analysis of lipid profile revealed no effect of training, supplementation, or supplementation and training interaction.

## 4. Discussion

The main finding of the present study was the rise of sIgA levels at rest after 90 days of Asx supplementation which was accompanied with a decrease in prooxidant-antioxidant balance (PAB). Also, the increase in neutrophil count and hs-CRP level recorded in placebo group was not apparent in supplemented group, indicating a significant blunting of the systemic inflammatory response in the subjects taking Asx.

The observed increase in sIgA levels after supplementation could indicate the effect of Asx on sIgA synthesis. Previous studies reported modulatory actions of Asx on humoral immune response such as induced production of polyclonal antibodies G and M in murine spleen cells [14], increased IgG and IgM production in cats and dogs [16, 17], partially restored humoral immune response in old mice [23], and enhanced immunoglobulin production in response to T-dependent stimuli in human blood cells [15]. Although the mechanism of Asx action was not known, it was suggested that it enhanced the antibody production through release of IL-1*α* [14], which is one of major regulating factors in B cell differentiation [24]. The beneficial effect of Asx on mucosal immunity, also found in our study, might be explained by its antioxidant activity [25]. Immune cells are particularly sensitive to oxidative stress due to a high percentage of polyunsaturated fatty acids in their plasma membranes, and they generally produce more oxidative products. Overproduction of ROS can tip the oxidant/antioxidant balance, resulting in destruction of cell membranes, proteins, and DNA [13]. ROS overproduction during physical activity might also inhibit locomotor and bactericidal activity of neutrophils, as shown in study of [26], where NK cell cytotoxic activity reduced the proliferation of T- and B-lymphocytes and promoted lymphocyte apoptosis.

Therefore, under conditions of increased oxidative stress (e.g., during disease states or physical activity), dietary antioxidants become critical in maintaining a desirable oxidant/antioxidant balance [27]. In our study, oxidative stress (measured by TOS) was significantly reduced after 90 days of regular training in both groups of soccer players, probably due to an upregulation in the body's enzymatic AO defense system. However, nonenzymatic antioxidants presented in plasma (measured by TAS) were not affected by training or supplementation during the observational period, which is in accordance with previous studies [28, 29]. Antioxidants are known to work synergistically to defend against oxidant production. In other words, when one AO nutrient is lacking in particular period of time, another could substitute it or it may be regenerated by another that is in abundance [30]. This may be the reason why no changes in TAS levels at rest were observed during 90 days of regular training and supplementation. Mobilization of tissue antioxidants into the plasma in order to maintain the AO status and to protect the body against ROS may also explain, in part, why no significant changes in TAS levels at rest were observed in response to training or Asx supplementation [31]. These results suggest that human organism has very potent mechanisms which protect cells against free radicals and oxidative stress.

However, we showed that Asx supplementation resulted in decreased prooxidant-antioxidant balance (PAB) during regular competitive season in young soccer players. Therefore, we believe that deleterious effects caused by the excess of ROS produced during physical activity can be additionally attenuated by the Asx. Asx induced a better redox balance favoring all cellular functions which depend on adequate amounts of ROS, thereby supporting the mucosal immune system. Our finding is in accordance with the study of Park et al. [18] who examined the possible immune-enhancing, AO, and anti-inflammatory activity of Asx in healthy women and showed that Asx could decrease a DNA oxidative damage and inflammation and enhance immune response.

The magnitude of muscle damage, evaluated by CK and LDH in our study, was reduced significantly over the observational period, probably by adaptation and conditioning of the muscle through regular training, as shown previously by Powers and Jackson [32]. Asx supplementation additionally attenuated exercise-induced muscle damage. Although muscle damage represents a direct consequence of exercise injury which mechanically disrupts muscle myofibrils, tissue damage associated to exercise-induced free radical production and subsequent oxidative stress was also suggested by Davies et al. [33]. It can be hypothesized that Asx protects the cell membranes against free radicals generated during heavy exercise, thus preserving the functionality of muscle cells. The unsaturated polygene chain of Asx could trap radicals in the membrane, while the terminal rings scavenge radicals both at the surface and in the interior of the phospholipid membrane [34]. Similar results were obtained in animal experiments, reporting that Asx attenuated oxidative damage of lipids and DNA in gastrocnemius and heart as well as the leakage of CK into plasma [35].

In this study, regular soccer training during the competitive season led to certain changes in leukocytes count only in placebo group. Over the time, total leukocytes count, neutrophil count, and hs-CRP levels had increased. The observed elevation in hs-CRP due to the regular intensive training was similar to the levels that have been associated with increased risk of coronary vascular disease [36]. These changes were not detected in supplemented group, which further supports the notion that Asx, as dietary supplement, has the ability to suppress minor inflammatory events induced by training. Anti-inflammatory action of Asx was previously detected in human and animal studies [16, 17, 35].

Evidence suggests that Asx has potential health-promoting effects in prevention and treatment of various diseases, such as chronic inflammatory diseases, metabolic syndrome, diabetes, cardiovascular disease, neurodegenerative diseases, exercise-induced fatigue, or male infertility [13], which likely involves AO mechanisms and decreased production of inflammatory mediators and cytokines. It is already known that physiologic stress, induced by prolonged and intensive physical activity, is reflected by transient, yet significant immune system perturbations, along with oxidative stress, muscle injury, and inflammation.

## 5. Conclusions

According to the results presented in this study, Asx supplementation improved sIgA response and attenuated muscle damage, probably due to restoring redox balance, thus preventing inflammation induced by rigorous physical training. Although our study focused on narrow population regarding the age, gender, and life style, nevertheless, it showed the modulating effects of Asx on the examined parameters. Our findings also indicate that Asx could show significant physiologic modulation in other individuals with mucosal immunity impairment or under conditions of increased oxidative stress and inflammation. Although the currently available data and recent findings are very encouraging, more extensive, well-controlled clinical trials are suggested for other threatened categories.

## Figures and Tables

**Table 1:** Subject characteristics at baseline.

	Astaxanthin	Placebo
Age (year)	17.9 ± 0.2	17.6 ± 0.1
Weight (kg)	71 ± 1.7	72 ± 1.8
Height (cm)	178 ± 1.4	180 ± 1.4
Body mass index (kg/m^2^)	22.4 ± 0.3	22.2 ± 0.4
Fat (%)	9.4 ± 0.7	9.7 ± 0.8
VO_2max_ (mL/min/kg)	55.5 ± 1.2	52.9 ± 0.7

Values are expressed as mean ± SE.

**Table 2:** Estimated daily energy and nutrient intake of soccer players.

	Asx	P
Energy (kcal)	3154 ± 247	2932 ± 147
Protein (g)	124 ± 8.7	125 ± 6.3
Carbohydrates (g)	412 ± 33	366 ± 23
Monosaccharides (g)	109 ± 12	123 ± 18
Fiber (g)	13.2 ± 1.4	12.6 ± 1.5
Fat (g)	104 ± 9	101 ± 7
Saturated fat (g)	33.2 ± 3.5	29.9 ± 3.2
Cholesterol (mg)	328 ± 23	344 ± 38
Vitamin A (IU)	2312 ± 442	2120 ± 389
Vitamin C (mg)	149 ± 25	135 ± 30
Vitamin E (mg)	5.4 ± 0.7	6.1 ± 1.5
Copper (mg)	2.3 ± 0.4	1.92 ± 0.5
Iron (mg)	14.4 ± 0.9	15.1 ± 1.6
Manganese (mg)	4.9 ± 0.3	4.0 ± 0.9
Selenium (*μ*g)	187 ± 15	164 ± 8
Zinc (mg)	13.0 ± 1.4	13.5 ± 1.8

Values are expressed as mean ± SE.

**Table 3:** Oxidative stress status of the soccer players at baseline and after 90 days of supplementation.

	Astaxanthin		Placebo		ANOVA		
	Before supplementation	After supplementation	Before supplementation	After supplementation	T	S	T × S
TAS (mmolTrolox equiv./L)	0.551 ± 0.036	0.538 ± 0.029	0.532 ± 0.039	0.585 ± 0.031	ns	ns	ns
TOS (mmol/L)	15.4 ± 1.5	5.0 ± 0.5^*∗∗∗*^	16.8 ± 1.6	5.1 ± 0.5^*∗∗∗*^	<0.001	ns	ns
PAB (HK U)	479.1 ± 45.4	291.5 ± 33.3^*∗∗∗*^	338.9 ± 48.8	258.9 ± 35.8	<0.001	ns	<0.05

Values are expressed as mean ± SE. ANOVA: T-training, S-supplementation, and T × S-training and supplementation interaction effect.

The difference in relation to baseline was significant at *p* < 0.001 (^*∗∗∗*^).

**Table 4:** Biochemical profile of the soccer players at baseline and after 90 days of supplementation.

	Astaxanthin		Placebo		ANOVA		
	Before supplementation	After supplementation	Before supplementation	After supplementation	T	S	T × S
AST (U/L)^†^	37.5 (29.2–48.1)	23.8 (20.1–28.1)^*∗∗∗*^	41.0 (35.7–47.0)	28.7 (24.5–33.6)^*∗∗*^	0.001	<0.05	ns
ALT (U/L)^†^	21.1 (16.8–25.6)	18.9 (15.6–21.6)	20.7 (18.6–22.5)	19.2 (16.1–22.6)	ns	ns	ns
CK (U/L)^†^	448 (330–608)	248 (172–360)^*∗∗*^	525 (283–974)	367 (244–552)	<0.01	ns	ns
LDH (U/L)^‡^	418.3 ± 13.8	303.5 ± 10.3^*∗∗*^	441.2 ± 17.5	359.4 ± 15.8^*∗*^	<0.001	<0.05	ns
UA (*μ*mol/L)^†^	330.3 ± 19.1	300.4 ± 14.0	346.5 ± 20.9	315.9 ± 15.5	<0.05	ns	ns
Cre (*μ*mol/L)^†^	129.6 ± 2.1	126.5 ± 2.2	133.1 ± 2.3	130.5 ± 2.4	<0.05	ns	ns
hs-CRP (mg/L)^†^	1.35 (1.00–1.82)	1.19 (0.89–1.59)	1.26 (0.89–1.78)	1.98 (1.24–3.17)	ns	ns	0.05
CHOL (mmol/L)^‡^	4.28 ± 0.20	4.39 ± 0.24	4.54 ± 0.28	4.50 ± 0.27	ns	ns	ns
HDL-C (mmol/L)^‡^	1.27 ± 0.19	1.30 ± 0.05	1.29 ± 0.07	1.31 ± 0.06	ns	ns	ns
LDL-C (mmol/L)^‡^	2.63 ± 0.19	2.73 ± 0.20	2.97 ± 0.24	2.91 ± 0.25	ns	ns	ns
TG (mmol/L)^‡^	0.84 ± 0.12	0.80 ± 0.08	0.95 ± 0.14	0.70 ± 0.09	ns	ns	ns

^‡^Mean ± SE.; ^†^Geometric mean values (95th confidence interval). ANOVA: T-training, S-supplementation, and T × S-training and supplementation interaction effect. The difference in relation to baseline was significant at *p* < 0.05 (^*∗*^), *p* < 0.01 (^*∗∗*^), and *p* < 0.001 (^*∗∗∗*^).

## Abbreviations

URTI: Upper respiratory tract infection
DTH: Delayed-type hypersensitivity
BMI: Body mass index
ELISA: Enzyme-linked immunosorbent assay
TAS: Total antioxidant status
TOS: Total oxidant status
PAB: Prooxidant-antioxidant balance
AST: Aminotransferase
ALT: Alanine aminotransferase
CK: Creatine kinase
LDH: Lactate dehydrogenase
UA: Uric acid
Cre: Creatinine
hs-CRP: High sensitivity C-reactive protein
CHOL: Total cholesterol
HDL-C: HDL cholesterol
TG: Triglycerides
LDL-C: LDL cholesterol
SE: Standard error
ROS: Reactive oxygen species
AMP: Adenosine monophosphate
ATP: Adenosine triphosphate.
